# ChatGPT’s advice is perceived as better than that of professional advice columnists

**DOI:** 10.3389/fpsyg.2023.1281255

**Published:** 2023-11-21

**Authors:** Piers Douglas Lionel Howe, Nicolas Fay, Morgan Saletta, Eduard Hovy

**Affiliations:** ^1^Complex Human Data Hub, Melbourne School of Psychological Sciences, University of Melbourne, Melbourne, VIC, Australia; ^2^School of Psychological Science, University of Western Australia, Perth, WA, Australia; ^3^Hunt Laboratory, University of Melbourne, Melbourne, VIC, Australia; ^4^Melbourne Connect, University of Melbourne, Melbourne, VIC, Australia

**Keywords:** ChatGPT, empathy, advice column, agony aunt, advice

## Abstract

ChatGPT is a high-performance large language model that has the potential to significantly improve human-computer interactions. It can provide advice on a range of topics, but it is unclear how good this advice is relative to that provided by competent humans, especially in situations where empathy is required. Here, we report the first investigation of whether ChatGPT’s responses are perceived as better than those of humans in a task where humans were attempting to be empathetic. Fifty social dilemma questions were randomly selected from 10 well-known advice columns. In a pre-registered survey, participants (*N* = 404) were each shown one question, along with the corresponding response by an advice columnist and by ChatGPT. ChatGPT’s advice was perceived as more balanced, complete, empathetic, helpful, and better than the advice provided by professional advice columnists (all values of *p* < 0.001). Although participants could not determine which response was written by ChatGPT (54%, *p* = 0.29), most participants preferred that their own social dilemma questions be answered by a human than by a computer (77%, *p* < 0.001). ChatGPT’s responses were longer than those produced by the advice columnists (mean 280.9 words vs. 142.2 words, *p* < 0.001). In a second pre-registered survey, each ChatGPT answer was constrained to be approximately the same length as that of the advice columnist (mean 143.2 vs. 142.2 words, *p* = 0.95). This survey (*N* = 401) replicated the above findings, showing that the benefit of ChatGPT was not solely due to it writing longer answers.

## 1 Introduction

ChatGPT, a groundbreaking artificial intelligence (AI) generative large language model (OpenAI, 2023), has recently garnered widespread attention due to its adeptness in various natural language processing tasks. Launched in November 2022, it experienced an unprecedented adoption rate, amassing over a million users in just 5 days and reaching 1.6 billion users by June 2023. Its creation marked a revolution in the industry, ushering in a new era of AI chatbots (Gohil, 2023).

It has also sparked significant interest within the academic community, leading to a wealth of scholarly literature (Kaddour et al., 2023; Ray, 2023). Illustratively, Katz et al. (2023) demonstrated that GPT-4 with zero-shot prompting could successfully pass the full United States legal Uniform Bar Exam, outperforming 90% of human participants. Similarly, Wu et al. (2023) showed that an enhanced version of GPT 3.5-Turbo could pass the Chinese Medical Licensing Examination, again surpassing the average human performance.

While ChatGPT’s technical prowess has been illustrated in various professional contexts, its capacity for nuanced human interactions remains an area of pivotal interest. Of particular interest is how well it can interact with humans in situations where it would need to convey empathy. Empathy plays a vital role in many domains (Hoffman, 2000; Sanders et al., 2021); if ChatGPT were to fail to exhibit sufficient empathy, this would adversely affect the quality of its interactions with humans (Leite et al., 2013). Indeed, numerous studies have argued that empathy is crucial for effective communication (Riess, 2017; Pounds et al., 2018; Janich, 2020) and that people are more persuasive when they appear to be empathetic (Lancaster, 2015). For reviews of the role of empathy in communication, please see Berger et al. (2010) and Floyd and Weber (2020).

The few studies that have explored the degree of empathy conveyed by ChatGPT reported that its responses often lacked empathy (Kalla and Smith, 2023; Sun et al., 2023; Zhao et al., 2023). GPT 3.5-Turbo performed poorly compared to the state of the art because it focused more on giving advice than addressing the user’s emotional needs (Zhao et al., 2023). Even GPT-4 was reported as having difficulty expressing empathy in a convincing fashion (Sun et al., 2023). However, these studies did not benchmark ChatGPT’s capabilities against those of humans.

It is necessary to compare ChatGPT to humans because if ChatGPT is perceived to perform worse than humans, it is likely that users will choose to interact with humans rather than with it. In a study reported in Ayers et al. (2023), human participants saw a series of medical questions that had been placed on Reddit’s r/AskDocs forum, the responses written by verified physicians and the responses written by GPT-3.5. Ayers et al. (2023) reported that participants rated the GPT-3.5 responses as being of higher quality than those of the physicians. A similar study was conducted by Liu et al. (2023) who compared physician response to 10 patient questions to the responses generate by GPT-3.5 and GPT-4. Liu et al. (2023) found that the responses by GPT-3.5 and GPT-4 were perceived as of higher quality than those written by the physicians.

One limitation of the above studies is that the physicians’ responses may not reflect typical doctor-patient interactions. Normally, doctors would spend some time explaining their diagnosis to the patient, ensuring that that the patient felt heard and respected. Conversely, the physicians’ responses in Ayers et al. (2023) were notably brief, averaging just 52 words, and sometimes as short as 17 words. Similarly, the physician responses in Liu et al. (2023) averaged 50 words and were sometimes as short as 20 words. In both studies, the physicians were focused on brevity and on conveying medical information, and not on addressing the emotional needs of the patient. It was therefore not appropriate to compare the empathy expressed in these responses to the empathy expressed in the responses by ChatGPT, as the physicians were often not attempting to be empathetic.

In our study, we assessed ChatGPT’s ability to provide advice in a situation where humans attempted to be empathetic. Specifically, we compared the responses of ChatGPT and humans to a series of social dilemma questions that had been submitted to a range of social advice columns (aka “agony aunt” columns). Our results suggest that ChatGPT can outperform humans in this domain.

## 2 Survey 1

### 2.1 Methods

We selected 10 newspaper advice columns: *Ask a Manager, Ask Amy, Ask E. Jean, Ask Ellie, Dear Abby, Dear Annie, Dear Prudence, Miss Manners, Social Q’s,* and *The Ethicist.* These columns were chosen because they were well-known and fielded a wide range of questions that we could access. For each column, we selected at random five questions. These questions were posted between November 2019 and June 2023. For each social dilemma question, we initiated a new chatbot session, ensuring that ChatGPT generated responses without any carryover context from previous questions. This was done using GPT-4 on the June 14, 2023. As we were interested in studying its default response, ChatGPT was not asked to be empathetic. For each question, we used the following prompt “Please respond to the following question [Social dilemma question text inserted here].” ChatGPT’s response and the response of the advice columnist were stripped of any identity-revealing information (e.g., “I am a chatbot” or “I am an advice columnist”). We always took ChatGPT’s first response. Both this and the subsequent study were approved the Human Research Ethics Committee at the University of Western Australia (2023/ET000523).

Participants in our study were each presented with just a single social dilemma question and the two answers (from the original advice column and from ChatGPT), without disclosing the origin of the answers. Thus, each of the 50 dilemmas were viewed, on average, by approximately eight participants. After viewing the question and corresponding answers, participants responded to a series of binary questions that evaluated the perceived quality of the answers provided.

In a series of binary questions, participants were asked which of the two answers was more balanced, more comprehensive, more empathetic, more helpful, and better. Following these assessments, we disclosed that one of the responses had been composed by a human and the other by a computer, and asked the participants to identify the computer-generated response. Finally, participants were asked to imagine a scenario where they had a question regarding a social dilemma and to indicate whether they would prefer this question be answered by a computer or by a human (i.e., a binary response).

To calculate an appropriate sample size for our study, we conducted a binomial power analysis (Champely, 2020). Assuming a significance level of 0.05 (two-sided), a null hypothesis of 0.5, and an alternative hypothesis of 0.6, the analysis revealed that we would require a sample size of 387 participants to achieve a statistical power of 0.8. This power level ensures a reasonably high probability of detecting a true effect if one exists. Based on this analysis, we decided to recruit 400 participants for the study.

Participants were recruited from Amazon’s Mechanical Turk (MTurk), a popular crowd-sourcing marketplace frequently used in psychological and behavioral research. To ensure the quality of data, we only recruited from a pool of MTurk workers who had previously been pre-screened to verify they were not bots. Additionally, our study was pre-registered to promote transparency and reproducibility in our research: https://aspredicted.org/66n24.pdf.

### 2.2 Results

**Figure 1:**
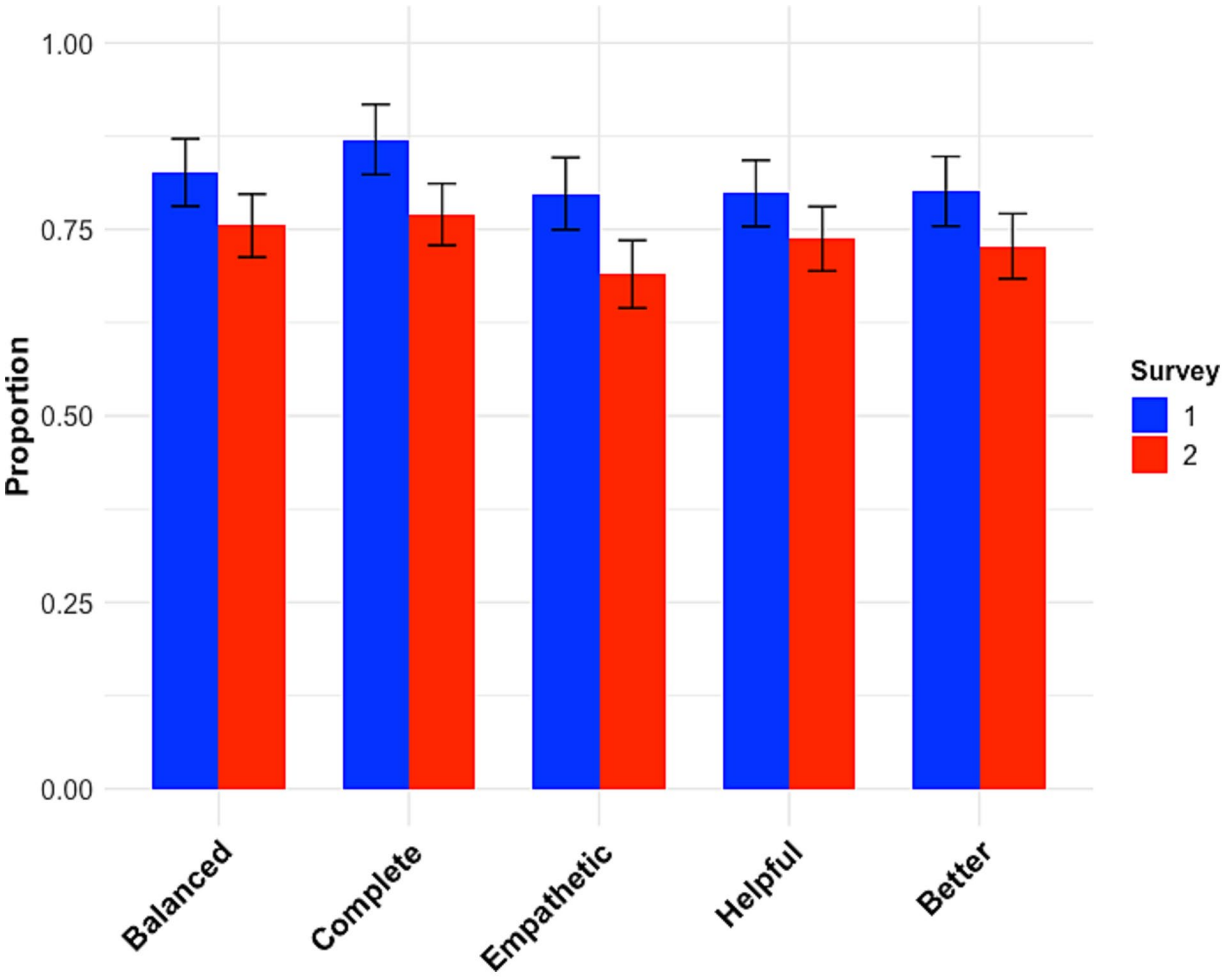
The proportion of participants who thought the answer provided by ChatGPT was more balanced, more complete, more empathetic, more helpful, and better than that provided by the professional advice columnist. **(A)** Survey 1. **(B)** Survey 2. Error bars represented 95% CI.

A total of 404 participants were recruited. Two were excluded as their data did not record properly, thereby preventing analysis. The data were analyzed using the tidyverse (Wickham, 2017), lme4 (Bates et al., 2015), lmerTest (Kuznetsova et al., 2017), purr (Henry and Wickham, 2021), and broom.mixed (Siegert, 2021) software packages in R (R Core Team, 2020). Participants’ mean age was 42.4 years (standard deviation = 12.1 years). The gender distribution was 156 female, 240 male, two non-binary, with four participants preferring not to disclose. The responses to the first five questions are depicted in Figure 1. Remarkably, for every question, ChatGPT clearly outperformed the professional advice columnists. Participants were not able to reliably identify which answer was written by the computer (only 54% succeeded). Despite this, the majority of participants (77%) indicated a preference for having their hypothetical social-dilemma questions answered by a human rather than by a computer.

In the pre-registration, we specified the use of binomial tests. However, post-experiment, we recognized that these tests failed to account for multiple subjects encountering the same social dilemma. To rectify this, we redid the analysis using a linear mixed-effects model, incorporating ‘dilemma’ as a random effect. Despite the binary nature of the dependent variables, we opted for linear models to gain unbiased estimates of our predictor variables’ causal effects (Gomila, 2021). The results of these statistical analyses are shown in Table 1.

**Table 1:** Statistical analysis for Surveys 1 and 2.

Question	Survey 1	Survey 2
Which answer do you think was more balanced?	*t*(49.9) = 14.1, *p* < 0.001	*t*(399) = 11.8, *p* < 0.001
Which answer do you think was more complete?	*t*(49.2) = 15.5, *p* < 0.001	*t*(399) = 12.8, *p* < 0.001
Which answer do you think was more empathetic?	*t*(48.3) = 12.1, *p* < 0.001	*t*(399) = 8.2, *p* < 0.001
Which answer do you think was more helpful?	*t*(47.1) = 13.2, *p* < 0.001	*t*(399) = 10.8, *p* < 0.001
Which answer do you think was better?	*t*(49.6) = 12.6, *p* < 0.001	*t*(399) = 10.2, *p* < 0.001
One of these answers was written by a computer. Which one do you think it was?	*t*(48.4) = 1.08, *p* = 0.29	*t*(399) = 0.5, *p* = 0.62
Assuming you had a social dilemma question and to get it answered you would need to put it in writing and receive a written response, would you prefer your question to be answered by a human or by a computer?	*t*(49.0) = 11.3, *p* < 0.001	*t*(399) = 19.0, *p* < 0.001

This table shows the results of the *t*-test for each question, for both Survey 1 and Survey 2. A statistically significant result shows that the proportion of participants choosing the ChatGPT answer over the human answer for that question in that survey was different from 50%. In other words, ChatGPT’s answer and the human answer were not equally preferable. Figure 1 shows the proportion of participants who preferred ChatGPT’s answer over the human answer.

Although not pre-registered, we also measured the word count for the official advice column answers and the answers written by ChatGPT. The word count for the official answers was considerably less than that for ChatGPT, with mean word count of 142.2 and 280.9 words, respectively. This difference was statistically significant, *t*(88.9) = 9.12, *p* < 0.001.

## 3 Survey 2

The second survey was identical the first survey except that, for each question, ChatGPT was requested to write an answer that was not longer than the official answer for that question. To do this, we used the following prompt: “Please respond to the following question in less than X words [Social dilemma question text inserted here],” where X was the word length of the official response. The survey was separately pre-registered: https://aspredicted.org/h5pk8.pdf.

A total of 401 participants were recruited. One was excluded because their data were corrupted. Participants’ mean age was 42.8 years (standard deviation = 12.5 years). The gender distribution was 187 female, 208 male, three non-binary, with two participants preferring not to disclose. While the ChatGPT answers were rarely exactly the same length as the corresponding official answer, on average they were very similar, with mean word counts of 142.2 and 143.2 words for the official answer and ChatGPT’s answer, respectively. This difference was not statistically different, *t*(97.7) = 0.06, *p* = 0.95.

As before, participants felt that the answers given by ChatGPT were more balanced, more complete, more empathetic, more helpful and better than the official answers (Figure 1; Table 1). As before, participants were not able to reliably identify the answer written by the computer (49% succeeded). Despite this, the majority of the participants (85%) indicated that if they had a social dilemma question, they would prefer it to be answered by a human.

Although we preregistered a mixed effects analysis with dilemma as a random effect, when we performed this analysis, R warned us that our fit was approaching a singularity. We therefore redid the analysis without dilemma as a random effect. The results of the second analysis are included in Table 1 and replicate what was found in the first analysis.

## 4 Discussion

Compared to the responses provided by advice columnists, ChatGPT’s responses were perceived as more balanced, complete, empathetic, helpful, and better. But participants were not able to determine which responses were generated by the computer at above chance levels. Despite this, when asked whom they would prefer to answer their own social dilemma question—a human or a computer—the majority of participants chose the human. Taken in aggregate, these findings show that ChatGPT outperformed the professional advice columnists, but that it was not the preferred choice among the participants, despite the fact its answers could not be distinguished from those of a human.

Though it is crucial for ChatGPT to deliver balanced, complete, and helpful answers, we were particularly interested in its ability to generate empathetic responses. Failing to do so could leave users feeling unheard and frustrated (Decety, 2011; Dalton and Kahute, 2016; Wu et al., 2023). While previous research has indicated that ChatGPT can provide more empathetic responses than doctors when the doctors were very brief and were not attempting to be empathetic (Ayers et al., 2023; Liu et al., 2023), to our knowledge, this is the first study demonstrating ChatGPT’s ability to surpass humans in displaying empathy in a situation where humans are attempting to do so.

As stated by Bellet and Maloney (1991), “Empathy is the capacity to understand what another person is experiencing from within the other person’s frame of reference, i.e., the capacity to place oneself in another’s shoes.” Empathy is typically expressed in written text via the so-called *interpersonal* channel (Halliday and Hasan, 1975), that is, in parallel to the main content and independent of the constraints of the medium. Producing empathetic language therefore requires the ability to calculate not only the phrasing of the primary (semantic) content but also the secondary (phatic, emotional, and interpersonal) content, and to interweave the two in a natural manner. Computational text generators in Natural Language Processing tend to be unable to do this; few generators have been able to produce text that communicates semantic and phatic content effectively (Duerr and Gloor, 2021). The ability of ChatGPT to emulate empathy is therefore all the more surprising, and calls for thorough investigation.

Recently, Belkhir and Sadat (2023) found that inserting into the prompt a statement about the system’s or the user’s emotional state affects the output produced. When the prompt contains “Looks like you are feeling <emotion>” the output contains more emotion-laden content, while when it contains “Try to understand how I am feeling,” it contains less. Why it does so is unclear. They measured the degree of emotionality of various kinds in the user input using the Electra classifier (Clark et al., 2020) trained on the GoEmotions dataset (Demszky et al., 2020) with 28 emotion labels.

Similar to both Ayers et al. (2023) and Liu et al. (2023), in our first survey we found the responses generated by ChatGPT were lengthier than those provided by the advice columnists. An appropriate response length is crucial for effective communication; an excessively long response could bore the reader, while an overly brief one might come across as curt and lacking empathy. In the first survey, we did not impose any word limit on ChatGPT’s responses, as we believe its determination of an appropriate response length was integral to the task. However, in the second survey we requested that, for each question, ChatGPT write an answer shorter than the official answer to that question. ChatGPT was largely able to do this and the average length of the ChatGPT answers was almost identical to the average length of the official answer. Despite this constraint, the second survey replicated the previous survey’s findings.

Contrary to the findings of Nov et al. (2023), in our study, participants could not distinguish ChatGPT’s responses from those written by a human, at least in this highly constrained setting. Furthermore, when blinded to the source of the answer, participants thought the answers produced by ChatGPT were better than those produced by humans. Despite this, most participants still preferred to have their social dilemma questions answered by a human than by a computer. This finding is consistent with a previous study that also found that humans prefer human-created responses (Reardon, 2023). It should be emphasized that in our study participants were not able to identify which answer was written by the computer and were not told which one was. Given that participants generally preferred the answers written by ChatGPT, had they been informed which answer was written by ChatGPT, they might have been more willing to have their own social dilemma questions answered by ChatGPT, rather by a human. Future research would need to investigate this issue.

## Data availability statement

Data, materials and analysis code (in R) can be found at https://osf.io/p5s2r/.

## Ethics statement

The studies involving humans were approved by Human Research Ethics Committee at the University of Western Australia (2023/ET000523). The studies were conducted in accordance with the local legislation and institutional requirements. The participants provided their written informed consent to participate in this study.

## Author contributions

PH: Conceptualization, Data curation, Formal analysis, Investigation, Methodology, Writing – original draft, Writing – review & editing. NF: Project administration, Writing – review & editing. MS: Writing – review & editing. EH: Conceptualization, Writing – review & editing.
